# MWCNT-Coated Glass Fabric/Phenol Composite Heating Panel Fabricated by Resin Infusion Process

**DOI:** 10.3390/polym15163353

**Published:** 2023-08-10

**Authors:** Seongpil Choi, Juyeop Park, Donghoon Kang, Sang-Eui Lee

**Affiliations:** 1 Department of Mechanical Engineering, Inha University, Incheon 22212, Republic of Korea; 2 Department of Mechanical Engineering, Hanyang University, Seoul 04763, Republic of Korea; 3 Railroad Accident Research Department, Korea Railroad Research Institute, Uiwang 16105, Republic of Korea

**Keywords:** carbon nanotube, glass fiber, resin infusion, dip-coating, heating performance

## Abstract

MWCNTs (multiwalled carbon nanotubes) were applied to fiber-reinforced composite materials with phenolic resin having flame retardance for the composite heating panels of railroad vehicles. Instead of dispersing MWCNTs in the matrix, the surface of a pristine plain-weave glass fiber fabric was coated with MWCNTs through a series of dip-coating and drying processes, followed by the resin infusion of the phenolic resin to make the composite heating panel. Before and after the resin infusion process, low percolation thresholds of 0.00216 wt%_MWCNT_ (weight percent of MWCNTs) and 0.001 wt%_MWCNT_, respectively, were achieved, as were very high electrical conductivities of 47.5 S/m at 0.210 wt%_MWCNT_ and 26.7 S/m at 0.116 wt%, respectively. The low threshold and high conductivity can be attributed to the formation of electrical pathways directly onto the glass fabrics. It was confirmed that mechanical properties such as modulus, strength, and maximum strain were at the same level as those of the pristine glass fabric composite. The heating performance with temperature uniformity, as well as the electrical and mechanical properties, indicates that the resin-infused glass fabric composite having MWCNTs directly coated onto the fabric surface can be a solution for lightweight structural composite heating panels for railway vehicles.

## 1. Introduction

Glass fiber (GF) is a reinforcing fiber mainly used in fiber-reinforced plastic (FRP) and has high specific strength, high environmental resistance, high chemical resistance, low thermal conductivity, low electrical conductivity, and a low dielectric constant [1,2,3]. It is used as structural material for aircraft fuselages [4], vehicle panels [5], pressure vessels [6], and boat hulls [7]. It is also used to make boards for electronic circuits [8] and blades for wind power generators [9,10], too. GF is relatively elongated and inexpensive compared to carbon fiber (CF), so its application is expanding in various fields, and related application research [11] including non-destructive technology [12] is also being actively conducted.

When CNTs (carbon nanotubes) are embedded in an appropriate amount in a composite material, the mechanical and electrical properties of the composite material can be improved [13,14,15,16,17,18,19,20,21,22,23]. However, when the content is higher than a certain level, the viscosity of the resin increases rapidly. In addition, voids can be generated in the process of resin infusion. During the curing of the composite material, water or solvents contained in the resin are evaporated and trapped in the resin due to its high viscosity [18].

Directly coating CNTs on the glass fibers rather than dispersing them in the matrix can be an appropriate solution to circumvent the above-mentioned resin viscosity increase and void generation. The grafting method has mainly been used by chemically modifying CNTs or glass fibers, and there have also been approaches using spraying, electrophoretic deposition (EPD), or chemical vapor deposition (CVD).

Zeng, et al. [19] used a commercially available multiwalled carbon nanotube (MWCNT) having a carboxyl group attached thereto. It was coated through chemical grafting using γ-aminopropyltriethoxysilane (APS), a silane coupling agent, and composited with resin transfer molding (RTM). Interlaminar shear stress (ILSS) and thermal conductivity were measured and compared, and the performance was improved by 40.5% and 55.3%, respectively, compared to the pristine glass fiber composite material. Tzounis, et al. [20] coated CNTs on glass fibers using two methods: physical adsorption and chemical grafting. Then, epoxy was coated to evaluate the enhancement in interfacial shear stress (IFSS). When the physical adsorption method was used, the IFSS was improved by only 13.4%, while the CNTs coated through grafting induced a 48% enhancement in the IFSS. The electrical conductivity of single fibers was also evaluated, with the results showing 4.4 S/m for the adsorption method and 14.2 S/m for the grafting method, respectively. Eskizeybek, et al. [21] silanized GFs through APS, and they also oxidized CNTs by using sulfuric acid to induce chemical grafting. Using the RTM process for composite plates, toughness was compared for four cases with or without CNTs applied to epoxy and fiber, respectively, and the case where CNTs were introduced to both resin and fibers showed 57% improvement in physical properties compared to the case where neither was applied.

Gnidakouong et al. [22] prepared a composite material by coating MWCNTs on GFs through the spraying process and impregnation with polyester resin through RTM. The electromagnetic wave shielding performance was measured up to 35.1 dB at 751 MHz. An, et al. [23] coated CNTs onto glass fiber fabric using EPD. In order to enable cathodic EPD, the CNTs were modified using ozone and polyethyleneimine (PEI), and then the coated GFs were impregnated with epoxy. The shear strength was maximized at 16 Vol%, and the increase in the physical property was 80% compared to the pristine fibers. Because EPD was used, a two-micrometer-thick CNT coating was obtained, which was difficult to achieve with dip-coating or spraying, which require a low-viscosity solution. He, et al. [24] used the CVD process to coat CNTs on GFs. CNTs grew vertically on the surface of the glass fibers and were aligned well. Epoxy was applied to the coated GFs and cured via hot pressing. The electrical conductivity of the fabricated composite material was evaluated according to the CNT mass fraction, and it was found to have an electrical conductivity of up to 100 S/m at the amount of CNT of 7 wt%.

As described above, although CNTs are coated with the various methods and properties were evaluated, GF fabric/phenol composites with CNTs directly coated by dip-coating have not been studied for heating applications.

In this study, MWCNTs were dispersed in an aqueous solution with a surfactant. MWCNT coating was conducted on GF fabrics by a series of dip-coating and drying processes, a simple and facile method. After laminating the coated glass fabrics, the vacuum-assisted resin infusion method (VARIM) was conducted with flame-retarding phenolic resin as the matrix material; the physical and mechanical properties, such as tensile and flexural properties, electrical properties, and heating performance were measured; and the effect of resin infusion was also monitored in order to investigate the feasibility of the multifunctional composite as a structural heating panel for transportation vehicles.

## 2. Materials and Methods

### 2.1. Fabrication of MWCNT-Coated GF Fabric

**Figure 1:**
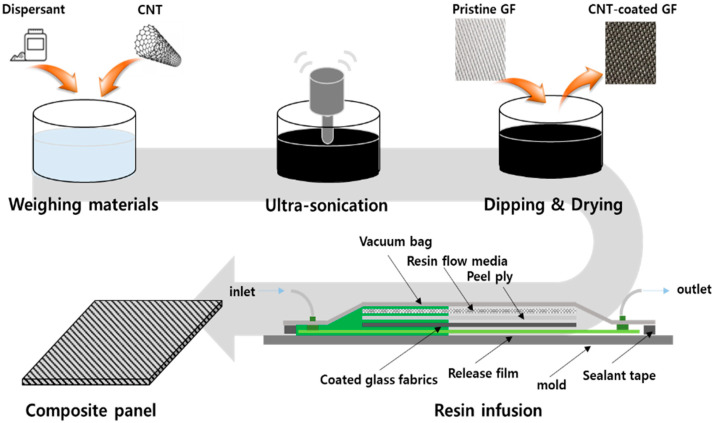
Schematic of dipping–drying process and resin infusion process for MWCNT-coated composite panel.

The overall process is shown in Figure 1. At first, in order to prepare a coating solution, MWCNTs, JENO 8A (JEIO Co., Incheon, Republic of Korea) and surfactant sodium dodecylbenzensulfonate, SDBS (tech grade) (Sigma Aldrich, St. Louis, MO, USA) were used. The mixing ratio of MWCNTs and SDBS varied from 1:1 to 1:20. An amount of MWCNTs of 1.0 g and 1 L of distilled water were employed, and the amount of SDBS was adjusted. GF fabrics with a size of 20 × 40 mm^2^ were coated for measurement of electrical conductivity.

As a result, 0.1 wt% of MWCNT and 1 wt% of SDBS were dispersed in 1 L of distilled water, and a tip-type ultrasonicator, VCX-750 (SONICS Co. Ltd., Newtown, CT, USA) was used for MWCNT dispersion for one hour with a period of a ten-second pulse and five-second pause at 500 W power.

**Figure 2:**
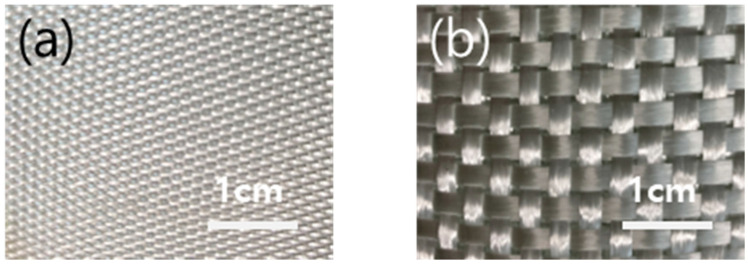
Glass fabrics of (**a**) yarn cloth (#823) and (**b**) roving cloth (#580).

Two types of as-received GF fabrics were used: E-glass fiber yarn cloth, #823, and roving cloth, #523 (DCT Co., Busan, Republic of Korea), as shown in Figure 2. The GF fabrics were cut to a size of 150 × 150 mm^2^ before coating, and a maximum of 20 cycles were performed with 1 cycle of dipping in the MWCNT solution and then drying in vacuum. The final drying process was carried out by vacuuming at 120 °C for 20 min.

### 2.2. Fabrication of MWCNT-Coated GF Fabric/Phenolic Resin Composite

A composite panel with the MWCNT-coated GF fabrics was prepared via VARIM [25]. The order is as follows. At first, a release film was laminated on a flat aluminum mold. Then, three sheets of the coated yarn cloth and six sheets of coated roving cloth were laminated, in order, as shown in Table 1, [823_1_/580_3_/823_1_/580_3_/823_1_], where the subscript is the number of plies.

Peel plies and resin flow media were layered onto the fabrics, and then the spiral tube for the resin guide was placed in the two opposite edges of the stacking and infusion plugs at the resin inlet and outlet were installed. The vacuum bag on the top was placed using sealant tapes. The vacuum pump was connected with the resin outlet so that the phenolic resin could flow into the inlet. After holding the vacuum once, the mold was heated up to 40 °C, a steady-state temperature. Flame-retardant phenolic resin was used, and the main agent, KRP-25L and hardener, KPH-L770 (Kolon industries Co., Seoul, Republic of Korea) were mixed in a ratio of 10:1. After air bubble removal for 10 min, the resin was injected through the resin inlet. When the resin was sufficiently injected, the valves at the resin inlet and outlet were closed to prevent additional flow of the resin, and curing was performed for four hours. The fully cured composite panel was demolded after cooling.

### 2.3. Characterization

Microstructures of uncoated and MWCNT-coated glass fiber fabrics were observed via SEM, S-4300SE, (Hitachi Co., Ltd., Tokyo, Japan) to evaluate uniform MWCNT coating on the glass fibers. Tensile and bending tests were performed with a universal testing machine for the composite panels made of glass fiber fabrics without and with CNT coating. ASTM-D638 and ASTM-D790 were referenced in accordance with the test standards of general fiber-reinforced composite materials.

Thermalgravimetric analyzer (TGA), TG209F3 (NETZSCH Korea, Paju, Republic of Korea) was used to confirm the thermal decomposition characteristics of the coated glass fiber fabric, and the experiment was conducted in air atmosphere at a temperature increase rate of 10 °C/min.

In electrical conductivity measurements, the resistance of the coated glass fiber before resin infusion was measured using the four-probe method, and after the resin infusion, the resistance was measured using the two-point method through electrodes connected to both ends of the composite material. Electrical conductivity (*σ*) was obtained from the measured resistance (*R*), the cross-sectional area (*A*), and the distance between electrodes (*L*), *σ* = *L*/(*RA*). The electrode was made of silver paste, ACH35001 (Protavic Co., Daejeon, Republic of Korea) with curing at 95 °C for 30 min and 180 °C for one and a half hours.

For the heating performance, the manufactured composite material was tested through the electrodes at both ends, and the temperature was measured using a thermocouple (K-type, 1EA) and an IR camera, Testo 882 (Testo Korea Ltd., Seoul, Republic of Korea). The thermocouple was attached to the center of the sample, and an IR camera was used to measure the uniformity of heat generation.

## 3. Results and Discussion

### 3.1. Dispersion of MWCNTs

**Figure 3:**
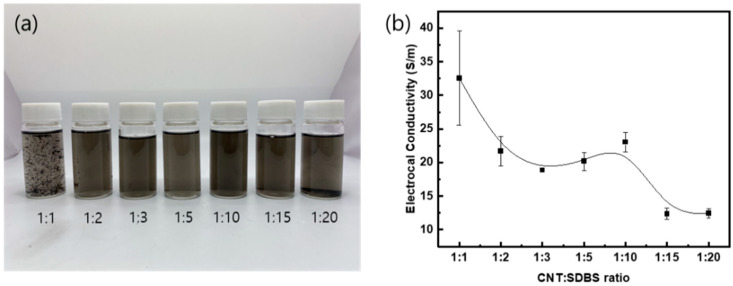
(**a**) MWCNT-dispersed water solution (diluted twenty times) and (**b**) electrical conductivity of MWCNT-coated GF fabrics with the ten cycles of dipping and drying, according to the ratio of MWCNT/SDBS.

The degree of dispersion is shown in Figure 3a. The solution was diluted 20 times with distilled water for visualization of the degree of dispersion (the original is shown in Figure S1). The stabilized dispersion was observed in the range of the mixing ratio of MWCNT and SDBS, 1:3 to 1:15. In addition, the electrical conductivity was measured for GF fabrics with ten cycles of dipping and drying.

The ratio of the conducting filler to the surfactant is essential, and there is a range of good dispersion for a given mixing ratio of single-walled carbon nanotubes (SWCNTs) and the surfactant, sodium dodecyl sulfate (SDS) [26]. If the amount of the surfactant is relatively low compared to the filler amount, the dispersion is poor due to the van der Waals attraction between the fillers. If the amount of the surfactant is above a certain level, the surfactant forms a self-assembled surfactant aggregation, known as micelles, and dispersion becomes unstabilized again due to the depletion of attraction. The dispersion of the MWCNT/SDBS/water solution in this study was found to be consistent with the SWCNT/SDS case.

In addition, the electrical conductivity of GF fabrics was measured after ten cycles of dipping and drying. Figure 3b shows that the conductivity decreased overall as the mixing ratio increased with a plateau at around 1:5 and 1:10 MWCNT/SDBS ratios. It is known that a higher electrical conductivity does not guarantee a better dispersion [27,28]. For example, shear manipulation of the suspension can induce an electrical network between the conductive fillers, which leads to enhancement in electrical conductivity. Based on the observation of the dispersion degree (Figure 3a) and the electrical conductivity (Figure 3b), the mixing ratio of MWCNT and SDBS of 1:10, was chosen for further experiments.

### 3.2. Microstructures

**Figure 4:**
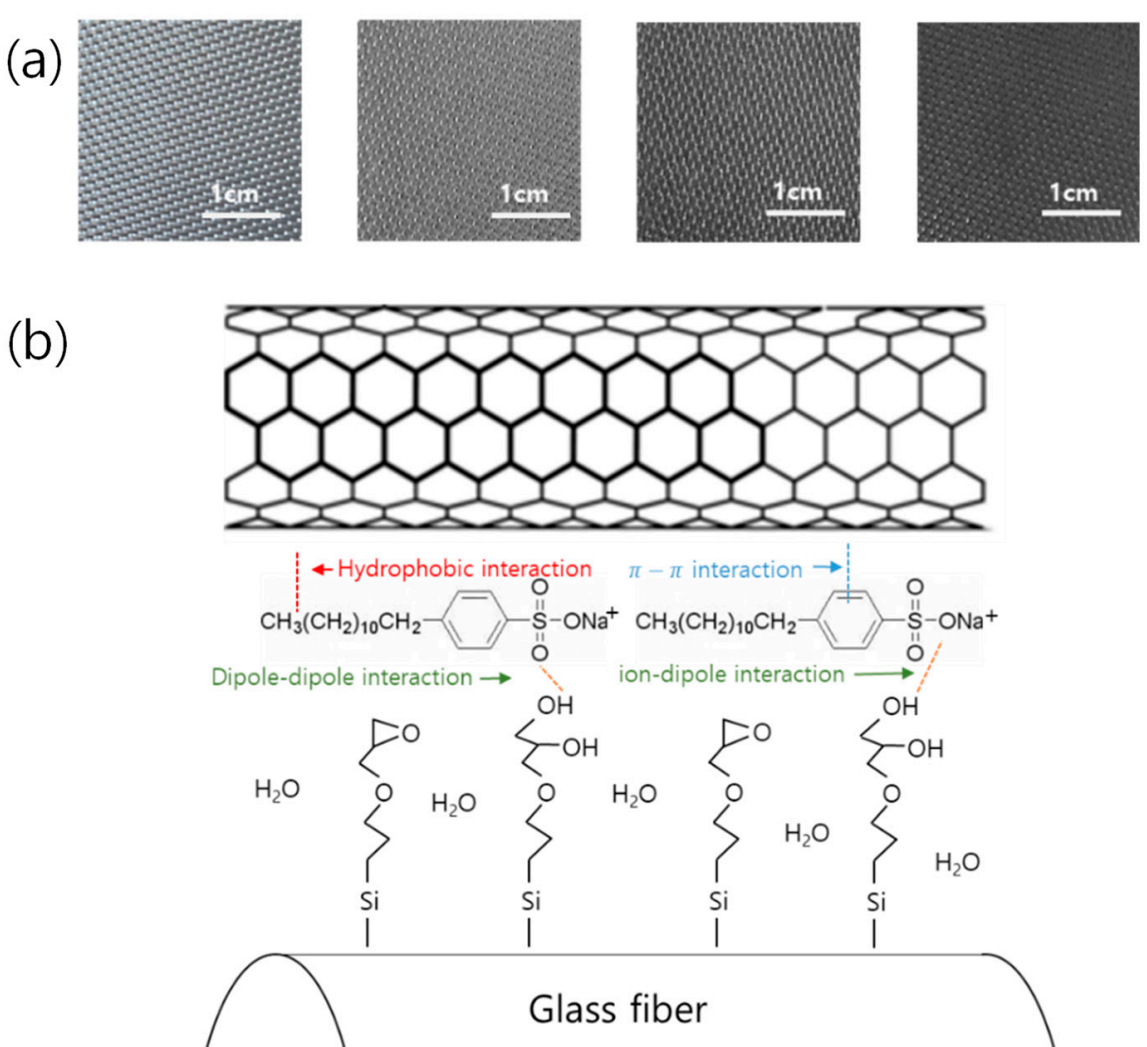
(**a**) Photographs of MWCNT-coated GF fabrics for 0, 1, 5, and 10 dipping–drying cycles; (**b**) schematic of interaction between MWCNTs-SDBS-GF fabric.

Figure 4a shows that the color changes with the increasing number of dipping–drying cycles (0, 1, 5, and 10 cycles). A uniform color was observed, and the color gradually became black, the color of the MWCNTs, as the number of cycles increased. Figure 4b is a schematic diagram of the interaction between the glass fiber, the SDBS, a surfactant, and the MWCNTs. The sizing agent coated on the GF surface has an epoxy group and reacts with water and SDBS, and a part of the epoxy group changes to a hydroxyl group to interact with the hydrophilic part of the SDBS [29]. Moreover, the hydrophobic heads of SDBS are linked to the surface of the MWCNTs through π–π bonds. As a result, the MWCNTs can be physiochemically bound to the glass fiber surface.

**Figure 5:**
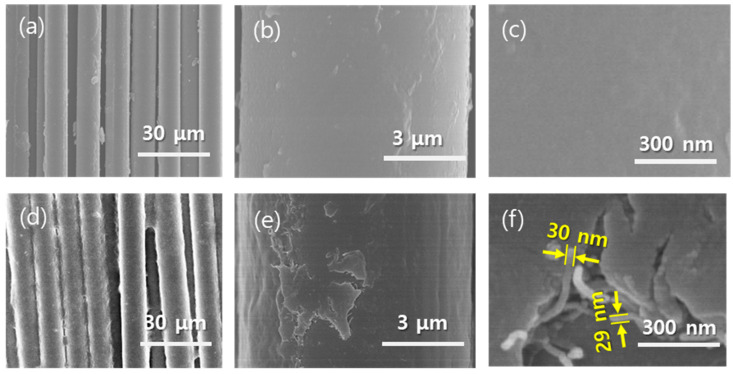
Microstructures of pristine GF fabrics (**a**–**c**) and MWCNT-coated GF fabrics of ten dipping–drying cycles (**d**–**f**).

The microstructure of the composite panel fabricated by the resin infusion process is shown in Figure 5. As seen in Figure 5a–c, the glass fibers have a smooth surface before coating. However, Figure 5d–f show that the MWCNTs are uniformly coated on and between the GFs. In Figure 5f, it can be confirmed that the MWCNTs are clearly observed on the GF’s surface, and the conducting fillers are densely and closely networked. The surface morphology is consistent with glass fibers coated with CNTs [24]. It can be expected that an electrical or thermal network will form along the surface of the glass fibers.

As can be seen in Figure 5f, the conducting fillers of 9 nm in diameter were observed to be around 30 nm in diameter via SEM, which is attributed to the SDBS coating and Pt sputtering. Pt coating for 120 s at 20 mA generated an 8 nm thick Pt coating. Therefore, the thickness of the SDBS on the MWCNTs is estimated to be 2.5 nm, and the corresponding volume of SDBS is calculated to be 142% of that of the MWCNTs. In the consideration of the densities of the MWCNTs (1.85 g/cm^3^) and the SDBS (0.18 g/mL), the mass fraction of MWCNTs by themselves can be estimated at 8.7 wt% in SDBS-covered MWCNTs.

**Figure 6:**
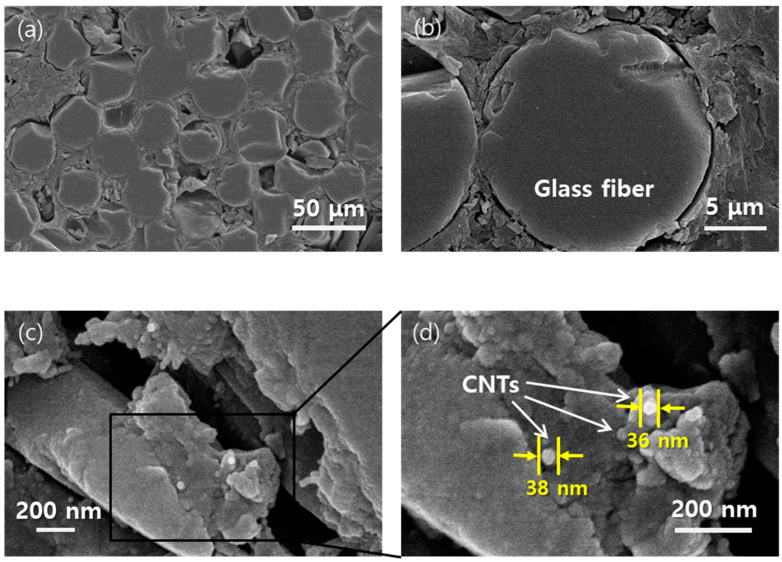
Microstructures of resin-infused MWNT-coated GF composite with ten cycles of MWCNT coating around glass yarn (**a**,**b**) and matrix region showing MWCNTs well covered by phenol (**c**,**d**).

Figure 6 shows the polished cross-section of the resin-infused MWCNT-coated GF composite panel. The phenolic resin is shown to be well-infused between the glass fibers in Figure 6a,b. In Figure 6b, a cross-section of a glass fiber shows that the resin covered the surface of the glass fibers. In Figure 6c,d of the region close to the surface of GF fabric yarn, MWCNTs are detected and observed to be well-covered by the phenolic resin.

### 3.3. Electrical Property

**Figure 7:**
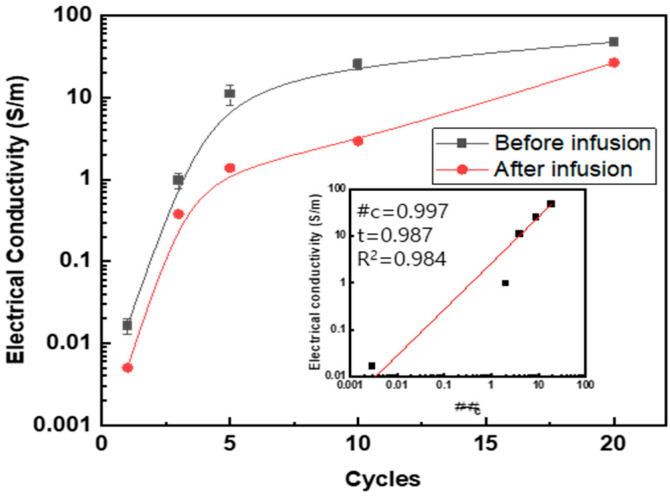
Electrical conductivity of MWCNT-coated GF fabric composites with regard to number of coating cycles.

**Figure 8:**
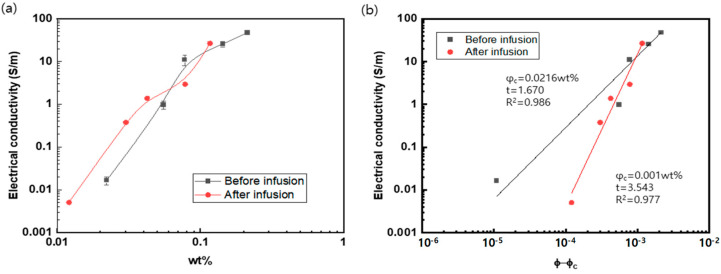
(**a**) Electrical conductivity of MWCNT-coated glass fiber composite in terms of mass fraction; (**b**) percolation threshold parameters before and after the resin infusion process.

The electrical conductivity with regard to the number of coating cycles was measured before and after the resin infusion process, as shown in Table 2, and Figure 7 and Figure 8. A percolation equation was proposed on the basis of the number of coating cycles. The formula is *σ* = *σ_0_* (*#*-*#_c_*)*^t^*, and the critical cycle, #_c_, is 0.997, indicating that an electrical network is formed with just one coating.

In Figure 7, the electrical conductivity after the resin infusion is also shown with the MWCNT weight fraction. After the resin infusion, the percolation behavior was observed. In addition, the electrical conductivity after resin infusion (26.7 S/m) at 20 cycles of dipping and drying was found to decrease compared to that before resin infusion (47.5 S/m), because the electrically insulating phenolic resin penetrates into the gap between the MWCNTs, and electron tunneling between the MWCNTs is impeded.

The electrical conductivity is found to be higher than or comparable to the values obtained in other studies [20,24,30,31], which means that the combination of MWCNT-SDBS onto GF works well for electrical networking. GF surfaces with CNTs chemically grafted on had an electrical conductivity of 20 S/m [20]. Compared with the results of other studies, the value of 20 S/m is similar to the case when 3 wt% of MWCNTs was coated on the fiber’s surface [24]. Another nanocarbon coated on the surface of glass fibers showed the same order of electrical conductivity with a maximum of 50 S/m at a synthesis temperature of 900 °C [30]. In addition, graphene coated on glass fiber had an electrical conductivity of 0.05 S/m [31].

The electrical conductivity as a function of the mass fraction is shown in Figure 8. For this purpose, the coated mass of the respective MWCNTs and SDBS was calculated by measuring the increment in mass with the number of coating cycles, together with the information of the MWCNTs’ diameter (9 nm) and the coating thickness of the SDBS (5 nm). The percolation parameters are presented in the inset graph.

Before infusion, the critical percolation threshold and the exponent were calculated as 0.0216 wt% and 1.670, respectively. The values are found in a very low level of the critical percolation fraction of 0.0021~5.0 wt% and the exponent of 1.2~3.2, respectively [27]. The electrical conductivity after the resin infusion reached 26.7 S/m at the MWCNT content of 0.116 wt%, one of the highest levels among CNT/polymer composites [27]. Moreover, the critical mass fraction after resin infusion was calculated as 0.001 wt%, which is lower than 0.0021 wt% and 0.0025 wt% found in the previously reported literature [27,28], where CVD-grown aligned CNTs with a diameter of 50 nm and an aspect ratio of 340 were dispersed in an epoxy matrix. This low critical mass fraction of 0.001 wt% may be attributed to the formation of a relatively long CNT network along the surface of the glass fiber fabric.

### 3.4. Mechanical Property

**Figure 9:**
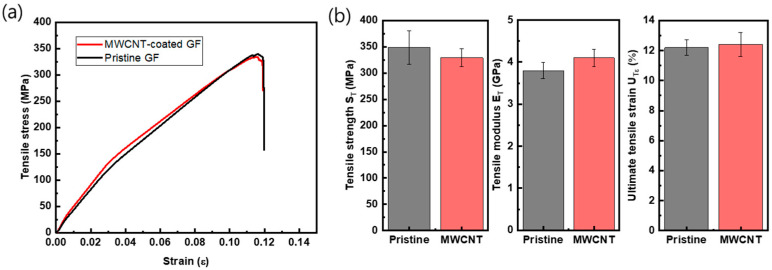
(**a**) Representative tensile stress–strain curves and (**b**) tensile properties of pristine and MWCNT-coated GF/phenolic resin composites (ten cycles of MWCNT coating).

**Figure 10:**
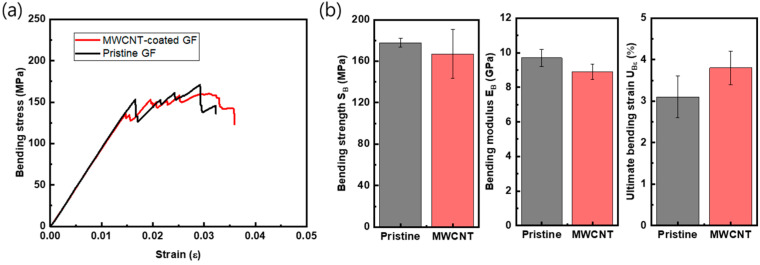
(**a**) Representative bending stress–strain curves and (**b**) bending properties of pristine and MWCNT-coated GF/phenolic resin composites (ten cycles of MWCNT coating).

Based on the required electrical conductivity, at least 20 S/m, for heating functions, MWCNT-coated GF fabric composites with ten dipping process cycles were chosen, and their mechanical properties were measured in tensile and flexural modes, which are shown in Figure 9 and Figure 10. Table 3 is the summary of these data. Figure 9a shows the graph of the tensile stress–strain curves of uncoated and coated GF fabric/phenol composites. Figure 9b is a summary of the strength (*S_T_*), modulus (*E_T_*), and ultimate strain (*U_Tε_*) of the MWCNT-coated GF/phenol composite with ten cycles of coating. The coated GF composite showed similar mechanical values as the pristine GF composite when the standard deviation (the error bars in the figures) is considered, with −5.4% in average tensile strength and +7.8% in average tensile modulus. In the case of the bending test, as shown in Figure 10, the average value of the modulus and the strength were slightly low compared to those of the pristine GF composite. However, in the case of the ultimate bending strain (*U_Bε_*), the MWCNT-coated composite had a value about 22.6% higher.

The mechanical properties of fiber-reinforced plastics with MWCNT reinforcement have been steadily investigated [14,15,16,17,18,19,21,23,32,33,34,35,36,37]. Enhancement in the mechanical properties can depend on various parameters: MWCNTs (length and diameter, surface chemistry, volume fraction, dispersion method in solvent and resin, alignment, etc.), resin (chemical structure, molecular weight, viscosity for impregnation, curing temperature, etc.), FRP (sizing agent, type of fiber preform (UD (unidirectional)), textile), MWCNT addition processing type (impregnation with MWCNT-dispersed resin solution, spraying, dipping, electrophoresis, CVD, etc.), composite processing (autoclave or vacuum bagging only with an MWCNT-added prepreg, resin infusion, resin transfer molding, and vacuum bagging only), and loading type (tension, compression, bending, shear, interlaminar shear), and environment in use (temperature, etc.) [14].

Any material combination (MWCNTs, resin, and FRP) together with processing and environmental conditions can be still seen as valuable in this research field due to the above-mentioned various parameters; it also means that researchers should be careful to extract a general tendency among the published data. Nevertheless, there are reports showing in Table S1 that MWCNTs help increase the matrix-dominant properties for bending and shear/interlaminar shear by retarding crack propagation and energy release on cracks [14,17,19,23,37]. For the tensile properties [16,19,21,37], there is an issue of the trade-off of the volume fraction of FRP and MWCNTs; that is, when MWCNTs are added above a critical level, the volume fraction of reinforcing fibers decrease due to the physical volume of MWCNTs, leading to an increase in the cured ply thickness [38]. In the viewpoint of glass fibers as primary reinforcement withstanding tensile loading and also the tensile properties directly proportional to the fiber volume fraction, the decrease in the fiber volume fraction can be fatal to increasing the tensile properties [37]. This is the reason why the addition of MWCNTs covered both negative and positive effects on the tensile properties (−36.0% [21] to +41.4% [19]), while it positively affected the matrix-dominated properties (+80.9%), even at the MWCNT content of 14 wt% [23].

The material combination employed in this study has not been reported, and this is the first observation conducted on the MWCNT-added GF/phenolic resin composite with resin infusion process. There are two aspects that can be emphasized: at first, in spite of the addition of the MWCNTs, the mechanical properties are observed to be fairly comparable to those of the pristine GF composites, as shown in Figure 9 and Figure 10. Secondly, the ultimate bending strain increased by 22.6%. This can be attributed to the strain energy release and mechanical bridging between MWCNTs hindering crack opening [15]. Therefore, the conclusion may be drawn that the MWCNT-coated GF/phenol composite with ten cycles of coating may be used as structural composite panel with a heating function embedded by the electrical conductivity of the MWCNT network on the GF fabrics.

### 3.5. Heating Characteristics

**Figure 11:**
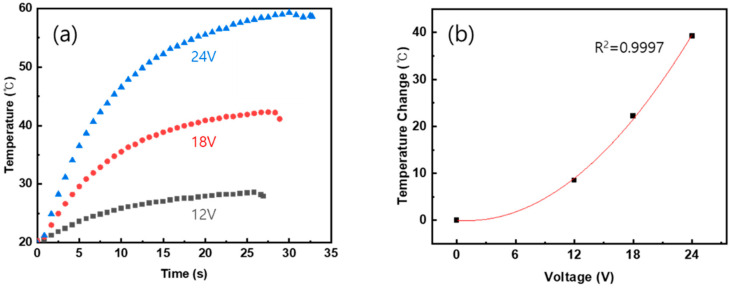
(**a**) Surface temperature of the composite panel with regard to applied voltage (12 V, 18 V, and 24 V) and (**b**) temperature change from room temperature to steady-state temperature and its quadratic fit.

The heating performance was measured using a thermocouple, and the results are shown in Figure 11. Figure 11a is a graph of temperature change over time when DC voltages of 12 V, 18 V, and 24 V are applied. In the end, the temperature reached 28.5 °C, 42.2 °C, and 59.3 °C for each applied voltage, and the temperature change according to the voltage is expressed as a graph in Figure 11b. Using Ohm’s law and heat capacity, the ratio of heat to voltage is expressed as: *Cm*∆*T* = *V*^2/*R* (where *C*, *m*, and *R* are the heat capacity, the mass, and the resistance of the composite, respectively, and *V* is the applied voltage). If the quadratic function is drawn in Figure 11b considering the temperature change and voltage, the coefficient of determination *R*^2^ is 0.9997, which is very close to the theory.

**Figure 12:**
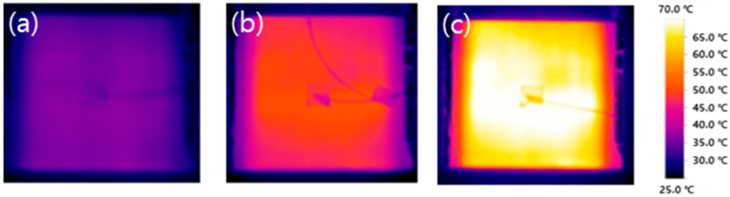
Thermal images of the fabricated composite panel with ten cycles of dipping–drying process for applied voltages at (**a**)12 V, (**b**) 18 V, and (**c**) 24 V.

Figure 12 shows the result of imaging the surface of the composite panel using an IR camera. Although the heat in the center is somewhat prominent, it shows a uniform temperature distribution overall. Therefore, it can be found that the MWCNT coating was uniformly formed on the GF fabric. In another study, when CNT-coated glass fiber was subjected to an electrothermal shock, even if the CNT-coated glass fibers were bundled, bent, and twisted, the heat was uniform and the temperature was kept constant [37].

**Figure 13:**
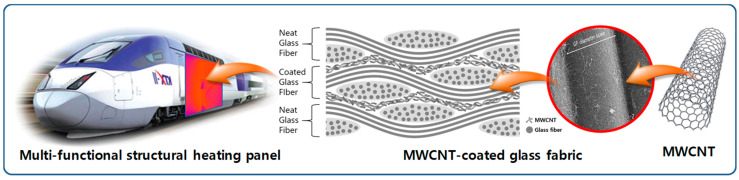
Schematic of MWCNT-coated glass fabric fabricated by resin infusion.

The schematic of this study is shown in Figure 13. Based on the electrical and mechanical properties and the heating characteristics, the conclusion may be drawn that the MWCNT-coated GF composite can be used as a structural heating composite panel.

## 4. Conclusions

In this study, MWCNTs were coated on glass fibers using the dip-coating method, which is a method of soaking glass fibers in a coating solution and drying them. It was confirmed that the coating was done well through observation of the microstructure using SEM. In addition, a composite material was produced using a resin infusion method using phenol resin. Electrical conductivity was evaluated, and when 20 coatings were performed, it was confirmed that the maximum conductivity was 47.5 S/m before resin infusion, and decreased by about half to 26.7 S/m after infusion. It is considered that the phenolic resin penetrated between the MWCNTs, interfering with the electrical network between the MWCNTs. As the number of coatings increases beyond a certain number of times, the mechanical strength deteriorates, so a composite material was produced with the minimum number of coated fibers having the required electrical performance of 10 S/m, and the mechanical strength of the pristine and coated glass fiber composites were evaluated. In the case of the ten-cycle MWCNT-coated glass fiber composite material, the mechanical strength showed slightly reduced performance, but the tensile stiffness increased by 7.8% and the bending strain by 22.6%. When compared with other studies, the results are shown to be reliable.

Heating performance was also evaluated. The applied voltages were DC 12 V, 18 V, and 24 V, reaching a maximum temperature of 28.5 °C, 42.2 °C, and 59.3 °C. In addition, it was possible to observe uniform heat generation on the surface of the composite material through the thermal image using the IR camera, and it is thought that it could be used as a heat-generating structure panel. As a result, we succeeded in manufacturing a MWCNT/GF composite material that maintains the structural performance with heating function.

## Figures and Tables

**Table 1:** Properties and stacking sequence of GF fabric panel.

Property	#823 Fabric	#580 Fabric
Type	Shaft satin	Plain weave
Thickness	0.23 ± 0.025 mm	0.6 ± 0.07 mm
Area density	294 ± 18 g/m^2^	580 ± 58 g/m^2^
Stacking sequence of layer	1, 5, 9	2, 3, 4, 6, 7, 8

**Table 2:** Electrical conductivity as a function of coating cycle and mass fraction.

Number of Cycles	Before Infusion		After Infusion	
	Weight Fraction (wt%)	Electrical Conductivity (S/m)	Weight Fraction (wt%)	Electrical Conductivity (S/m)
1	0.022 (±0.001)	0.0165 (±0.00343)	0.012 (±0.001)	0.0051 (±0.0001)
3	0.055 (±0.003)	0.978 (±0.206)	0.030 (±0.002)	0.379 (±0.017)
5	0.078 (±0.004)	11.0 (±3.00)	0.042 (±0.002)	1.38 (±0.053)
10	0.140 (±0.007)	25.4 (±3.70)	0.078 (±0.004)	2.95 (±0.12)
20	0.210 (±0.001)	47.5 (±1.53)	0.116 (±0.0005)	26.7 (±2.4)

**Table 3:** Mechanical properties of pristine and MWCNT-coated GF composite panels.

Property	Pristine GF Composite	MWCNT-Coated GF Composite	Increment
$S_{T}$(MPa)	348.5 (±31.7)	328.9 (±16.9)	−5.4%
$E_{T}$(GPa)	3.8 (±0.19)	4.1 (±0.21)	+7.8%
$U_{T\varepsilon }$	0.122 (±0.005)	0.124 (±0.008)	+1.6%
$S_{B}$(MPa)	177.8 (±4.2)	167.1 (±23.4)	−5.9%
$E_{B}$(GPa)	9.7 (±0.49)	8.9 (±0.45)	−8.3%
$U_{B\varepsilon }$	0.031 (±0.005)	0.038 (±0.004)	+22.6%

## Data Availability

Not applicable.

## Supplementary Materials

The following supporting information can be downloaded at: https://www.mdpi.com/article/10.3390/polym15163353/s1, Figure S1: MWCNT water solution with the ratio of MWCNT:SDBS (1:1, 1:3, 1:5, 1:10, 1:15, 1:20); Table S1: Studies on mechanical properties of MWCNT-added glass fiber-reinforced composites.
