# Supplementary material for: MWCNT-Coated Glass Fabric/Phenol Composite Heating Panel Fabricated by Resin Infusion Process
Source: Polymers (Basel). 2023 Aug 10;15(16):3353. doi: 10.3390/polym15163353 (PMC10459559; doi:10.3390/polym15163353)
Supplement: Supplementary file 1 [file polymers-15-03353-s001.zip › polymers-2020470-supplementary.pdf]

Supporting Information

MWCNT-coated glass fabric/phenol composite heating panel fabricated by resin infusion process

Seongpil Choi[1], Juyeop Park[2], Donghoon Kang[3,*], and Sang-Eui Lee[1,*]

[1] Department of Mechanical Engineering, Inha University

[2]Department of Mechanical Engineering, Hanyang University

[3] Railroad Accident Research Department, Korea Railroad Research Institute

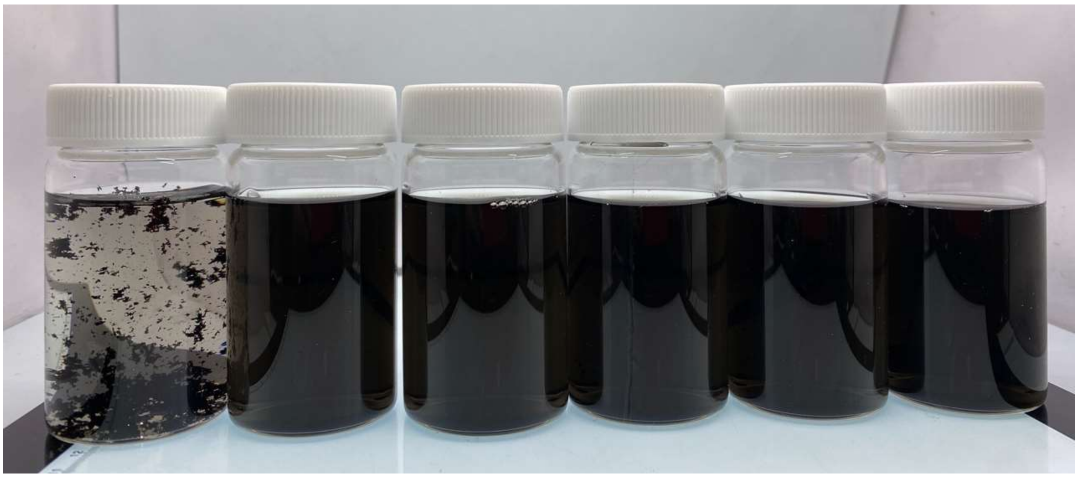

Figure S1. MWCNT water solution with the ratio of MWCNT:SDBS (1:1, 1:3, 1:5, 1:10, 1:15, 1:20)

In Figure S1, the undiluted CNT-SDBS solution is shown. As shown in the figure, the dispersion was not observed due to the thick dark color, except in the ratio of MWCNT/SDBS ratio of 1:1. Therefore, the solutions were diluted 20 times for the Figure 4(a). Multiple light scattering didn't work for measuring the change in the dispersion with time. Instead, the solutions were coated on GF fabrics and then the electrical conductivity was measured to compare the dispersion of MWCNTs according to the ratio of the filler and SDBS.

Table S1. Studies on mechanical properties of MWCNT-added glass fiber-reinforced composites

Ref #	CNT	Resin	FRP	CNT content	Tensile test	Bending test	Shear test	Property	Pristine FRP	CNT-added FRP	Increment	Note
14	MWCNT	epoxy	GF - UD	0.1 wt%		O		E_B	24.3	27.1	11.5%	
								S_B	317.0	420.8	32.7%	
								U_B	1.9	2.1	9.4%	
16	CNT	epoxy	GF - UD	0.3 wt%	O			S_T	430	475	10.5%	
17	Oxidized-MWCNT	epoxy	GF - fabric	1.0 wt%			O	*ILSS*	32.49	38.4	18.2%	
19	MWCNT-COOH	epoxy	GF - fabric	0.5 g/L	O	O	O	E_T	29	41	41.4%	
								S_T	615	810	31.7%	
								E_B	15.8	21.6	36.7%	
								S_B	1325	1725	30.2%	
								ILSS	56	81	44.6%	
21	Oxidized-MWCNT	epoxy	GF - UD	0.1wt%	O			E_T	7.5	7.25	-3.3%	Epoxy modification
								S_T	335	370	10.4%	
								E_T	7.5	4.8	-36.0%	Both modification on GF and epoxy
								S_T	335	325	-3.0%	
23	Ozone-treated MWCNT	epoxy	GF - UD	14%			O	*IPSS*	47	85	80.9%	
37	MWCNT	epoxy	GF fabric	0.4 wt%	O		O	E_T	22.2	23.4	5.4%	
								S_T	410.5	418.4	1.9%	
								ILSS	41.9	54.7	30.5%	

IPSS: In plane shear strength, *E*: modulus (GPa), *S*: strength (MPa). Subscript *B*: bending subscript *T*: tensile
